# An Improved Label-Free Indirect Competitive SPR Immunosensor and Its Comparison with Conventional ELISA for Ractopamine Detection in Swine Urine

**DOI:** 10.3390/s17030604

**Published:** 2017-03-16

**Authors:** Sai Wang, Shuai Zhao, Xiao Wei, Shan Zhang, Jiahui Liu, Yiyang Dong

**Affiliations:** Beijing Key Laboratory of Bioprocess, College of Life Science and Technology, Beijing University of Chemical Technology, Beijing 100029, China; lqfhws@163.com (S.W.); zhaoshuaibuct@163.com (S.Z.); weixiaobuct@163.com (X.W.); zhangshanbuctbuct@163.com(S.Z.); liujiahuibuct@163.com (J.L.)

**Keywords:** SPR immunosensor, ractopamine, swine urine, ELISA

## Abstract

Ractopamine (RCT) is banned for use in animals in many countries, and it is urgent to develop efficient methods for specific and sensitive RCT detection. A label-free indirect competitive surface plasmon resonance (SPR) immunosensor was first developed with a primary antibody herein and then improved by a secondary antibody for the detection of RCT residue in swine urine. Meanwhile, a pre-incubation process of RCT and the primary antibody was performed to further improve the sensitivity. With all the key parameters optimized, the improved immunosenor can attain a linear range of 0.3–32 ng/mL and a limit of detection (LOD) of 0.09 ng/mL for RCT detection with high specificity. Furthermore, the improved label-free SPR immunosenor was compared thoroughly with a conventional enzyme-linked immunosorbent assay (ELISA). The SPR immunosensor showed advantages over the ELISA in terms of LOD, reagent consumption, analysis time, experiment automation, and so on. The SPR immunosensor can be used as potential method for real-time monitoring and screening of RCT residue in swine urine or other samples. In addition, the design using antibody pairs for biosensor development can be further referred to for other small molecule detection.

## 1. Introduction

Ractopamine (RCT), a typical agonist belonging to the β-agonist family, was originally used as veterinary medicine for respiratory diseases and bronchodilators [1,2]; however, some unscrupulous manufacturers ignore the promulgated laws and regulations [3], and apply excess RCT to promote animal growth. The inducing RCT residue in animal-derived food has a potential risk for affecting the human cardiovascular and central nervous systems. Efficient supervision of RCT can greatly safeguard consumers’ health. RCT residue detection was traditionally performed with high-performance liquid chromatography (HPLC), liquid chromatography/mass spectrometry/mass spectrometry (LC–MS/MS), and immunoassays such as the enzyme-linked immunosorbent assay (ELISA) [4,5,6,7,8,9]. LC–MS/MS and HPLC obtain a limit of detection (LOD) of about 2 µg/kg [4,5,6], but require a complicated sample extraction process, a long time and a high cost. On the other hand, ELISA or fluorescence-based immunoassays for β-agonist detection allow LODs of less than 2 µg/kg with good specificity [7,8,9], but they take a long time due to incubation steps, require multiple washing steps as well as biological or chemical labels. It is important and urgent to develop efficient and simple analytical methods with high specificity and sensitivity for RCT residue detection.

Surface plasmon resonance (SPR) is one of the dominant analytical technologies for label-free detection of chemical and biological species [10,11,12]. The detectors measure refractive index (RI) changes induced by specific binding between the ligand and analyte, generate mass-dependent signals, and realize real-time monitoring. The SPR immunosensor is based on the specific antibody-antigen recognition, which also minimizes the matrix effect in the flowing system. Moreover, the SPR-based method can be label-free, which facilitates the further development of the immunosensor. Liu et al. [13] developed an indirect competitive SPR immunosensor for RCT detection in pork liver samples and obtained a LOD of 0.12 ng/mL and a narrow linear detection range of 0.28–4.29 ng/mL. We herein focus on the improvement of the LOD and linear range of the SPR immunosensor for RCT detection in swine urine samples, and we also developed an ELISA immunosensor for validation and comparison.

In brief, a sensitive and specific label-free SPR immunosensor was developed based on an indirect competitive assay first using a primary antibody, and then it was improved by introducing a secondary antibody for signal amplification. The improved SPR immunosensor improved the LOD from 0.22 ng/mL to 0.09 ng/mL, and provided a linear detection range of 0.3–32 ng/mL. Meanwhile, we applied a pre-incubation of RCT with the primary antibody before injection for the competition to ensure the prior recognition of RCT and the primary antibody. Deliberate optimization towards the coupling concentration and pH, the dilution ratio of antibodies, and the pre-incubation time was carried out. The label-free indirect competitive SPR immunosenor was then compared thoroughly with conventional ELISA in terms of LOD, reagent consumption, analysis time, and experiment automation.

## 2. Materials and Methods

### 2.1. Materials and Apparatus

Nhydroxysuccinimide (NHS), *N*-(3-dimethylaminopropyl)-*N*’-ethylcarbodiimide hydro-chloride (EDC), and ethanolamine were purchased from Sigma-Aldrich (Shanghai, China). Anti-RCT mouse monoclonal antibody (defined as antibody I) and anti-mouse rabbit antibody (defined as antibody II) were kindly provided by Beijing KWINBON Biotechnology Co., Ltd. (Beijing, China). RCT standards, RCT-BSA (Bovine Serum Albumin), HRP (Horseradish peroxidase)-labeled anti-mouse rabbit antibody (defined as HRP-antibody II) and negative swine urine samples were purchased from NanKai Biotechnology Co., Ltd. (Hangzhou, China). Bovine serum albumin (BSA) and 3,3′,5,5′-tetramethylbenzidine dihydrochloride (TMB) were purchased from KPL (Gaithersburg, MA, USA). Other chemical reagents were purchased from Beijing Chemical Works (Beijing, China). All chemicals were of analytical grade.

Buffers were prepared with ultrapure water and filtered using 0.22 μm membrane filter before use. Negative swine urine samples were centrifuged at 5000 rpm for 10 min and filtered with a 0.22 μm filter befor use.

SPR assay was operated on a Biacore T200 incorporating a CM5 chip purchased from GE Healthcare Life Sciences (Uppsala, Sweden). ELISA was performed in Flat-Bottom Immuno Plates (Nunc, Denmark). Plates were washed using *ELx50* microplate strip washer (Winooki, VT, USA) and absorbance was measured using *ELx800* absorbance microplate reader (Winooki, VT, USA) at 450 nm.

### 2.2. Indirect Competitive SPR Immunosensor

**Scheme 1:**
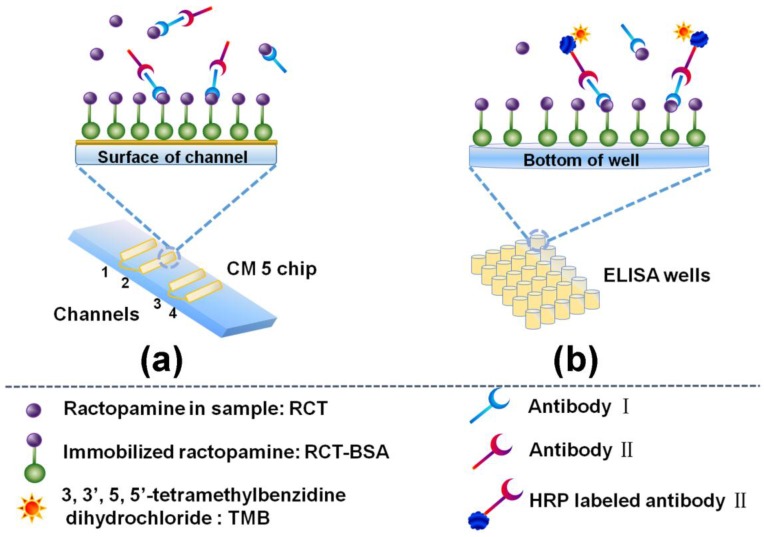
Schematic illustration of the label-free indirect competitive SPR immunosensor and the ELISA immunosensor. For both assays, RCT-BSA was immobilized on solid surface, RCT/antibody I mixture was added for competitive reaction, and antibody II/HRP-antibody II was added to bind with antibody I and used for signal amplification. (**a**) RCT-BSA was immobilized on channel 2 of CM5 chip, and the SPR signal responses were generated along with the combination of the RCT-BSA/antibody I/antibody II. Channel 1 was used as a reference channel; (**b**) RCT-BSA was immobilized on the bottom of microwells and the ELISA signals were obtain by the catalytic reaction of HRP and TMB.

Scheme 1a illustrates the developed SPR immunosensor, RCT-BSA was immobilized on the CM5 chip surface by amine coupling. Ethanolamine was then used to block the blank sites. The mixture of RCT/antibody I/antibody II was injected into the SPR flowing channel for competition. The specific binding of RCT-BSA/antibody I and antibody I/antibody II, caused RI changes and angle variation. The Biacore T200 transduced the RI change and angle variation to signal intensity with units defined as resonance units (RU).

The detailed assay protocol was described as following: The CM5 chip surface was activated with 0.4 M EDC/0.1 M NHS (1:1, v/v) at a speed of 10 µL/min for 7 min. Optimized concentration of RCT-BSA (with a conjugation ratio at about 6:1) in optimized buffer was injected into SPR flowing channel at a speed of 10 µL/min for 11 min to get an immobilization signal of 10,000 RU, and then the surface was blocked with 1 M ethanolamine flowing at 10 µL/min for 7 min to deactivate the excess sites, and this channel was defined as sensing channel. A control channel was treated simultaneously as a reference channel without RCT-BSA immobilization to measure the nonspecific adsorption. Different concentrations of RCT was mixed respectively with optimized dilution of antibody I (1:1, v/v) for optimized time and then injected with 1:9000 antibody II (2:1, v/v) into the SPR flowing channel at a speed of 20 µL/min for 4 min. After each injection, the CM5 chip was regenerated with 50 mM NaOH flowing at 20 µL/min for 1 min. PBS (10 mM, pH 7.4) was used throughout the assay. Signals were collected and two sensorgrams (RU vs. Time) were obtained for each sample in sensing channel and control channel. The final sensorgram was derived and charted from response difference between the two sensorgrams. Final signal of each sample was denoted by B_i_, and B_0_ was used for negative control (without RCT). The signal values were converted into corresponding % bound relative to the blank (B_i_/B_0_, *%*).

#### Optimization of Assay Conditions

Firstly, pre-concentration was carried out with different concentrations of RCT-BSA (5, 10, 50, and 100 µg/mL) flowing at a speed of 10 μL/min for 2 min, and after each injection, the absorbed RCT-BSA was removed by 50 mM NaOH flowing at 20 µL/min for 1 min. With an optimized concentration, four kinds of acetate buffers with different PH (4.0, 4.5, 5.0, 5.5) were used to optimize the coupling PH, and the absorbed RCT-BSA was removed by 50 mM NaOH flowing at 20 µL/min for 1 min. With the optimized conditions, the CM5 chip was functionalized with RCT-BSA, blocked with ethanolamine, and was ready for use.

Secondly, dilution of antibody I needs to be evaluated, because insufficient antibody would result in weak SPR signal and narrow detection range, and too much antibody would affect the sensitivity of the immunosensor. Thirdly, as antibody I was competed by RCT and RCT-BSA, pre-incubation time of RCT with antibody I was optimized to ensure the best prior recognition of RCT and antibody I.

LOD was defined as the RCT concentration corresponding to three standard deviations below the mean signal from negative control.

### 2.3. Indirect Competitive ELISA Immunosensor

A conventional indirect competitive ELISA was developed at the same time for validation and further comparison for RCT detection in swine urine. As is shown in Scheme 1b, RCT-BSA was immobilized on the bottom of the microwells and unbound RCT-BSA were removed by washing, then BSA was used for blocking. The RCT in samples and antibody I were pre-incubated and then added into the microwells. The competition was occurred among the RCT, RCT-BSA and the limited antibody I. After removing the excess antibody I and RCT, HRP-labeled antibody II was used for signal amplification as the HRP catalyzed TMB substrate for colorimetric reaction.

The detailed protocol was described as following: The wells were firstly coated with 100 µL/well of 5 μg/mL RCT-BSA in 50 mM bicarbonate buffer (pH 9.6), and incubated overnight at 4 °C. Wells were washed three times with 300 µL/well of wash buffer PBS-T (10 mM PBS, pH 7.4, 0.05% Tween-20) to remove the unbound RCT-BSA, and were then blocked with 200 µL/well of 2% BSA/PBS (w/v) for 2 h at 37 °C and the unbound protein was removed. Subsequently, 100 µL/well pre-incubated 1:1000 antibody I and various concentrations of RCT (1:1, v/v) in solution were added and the binding was allowed for 45 min with mild shaking. After washing, 100 µL/well 1:9000 HRP-labeled antibody II was added, and the incubation was accomplished after 45 min at 37 °C. Finally, 100 µL/well TMB solution was added and incubated for 20 min at 37 °C until the color of solution changed from colorless to blue. Then the color reaction was stopped by adding 50 µL of 2 M H_2_SO_4_ and the color turned to yellow. The absorbance was measured immediately at 450 nm. Each step was protected from light. The final absorbance of the competition system, denoted by B_i_, was consistent with the result of absorbance at 450 nm. B_0_ means the absorbance value of the negative control (without RCT). The absorbance values were converted into their corresponding test % bound relative to the blank (B_i_/B_0_, %).

## 3. Results

### 3.1. Optimization of the RCT-BSA, Antibody I Dilution, and Pre-Incubation Time of RCT and Antibody I

**Figure 1:**
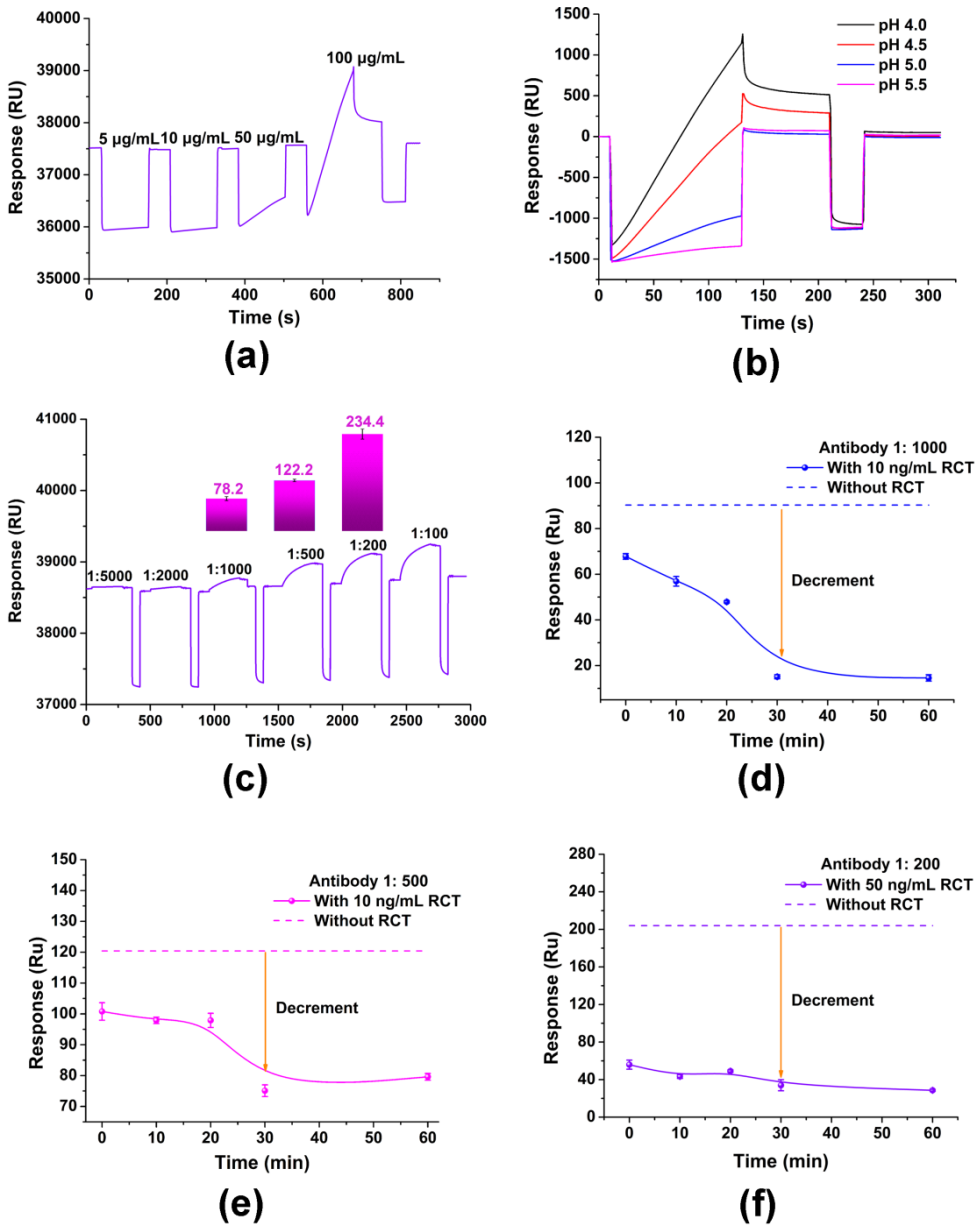
Optimization of key parameters of the primary SPR immunosensor. (**a**) Optimization of the coupling concentration of RCT-BSA; (**b**) Optimization of the coupling pH of RCT-BSA; (**c**) Optimization of the dilution ratio of antibody I; (**d**) Optimization of pre-incubation time with antibody I diluted at 1:1000; (**e**) Optimization of pre-incubation time with the antibody I diluted at 1:500; (**f**) Optimization of pre-incubation time with the antibody I diluted at 1:200. Data points are the average ± standard deviation (*n* = 3).

RCT-BSA is the one of the key elements in the competitive reaction. Insufficient RCT-BSA will result in weak signals and excess RCT-BSA may affect the sensitivity of the immunosensor. As is shown in Figure 1a, different concentrations of RCT-BSA (5, 10, 50, and 100 µg/mL) were evaluated. The pre-concentration was based on electrostatic adherence; thus, the condition that caused a sharp but not saturated responsive SPR signal was more preferable, and 100 µg/mL was used for subsequent assays. Similarly, acetate buffers with different pH values (4.0, 4.5, 5.0 and 5.5) were tested with 100 µg/mL RCT-BSA, and pH 4.0 was set for further immobilization (Figure 1b). With the optimized condition, the CM5 chip was functionalized following the protocol described above in Section 2.2, and was ready for use.

Antibody I is the dominant factor for the linear detection range and the sensitivity, because insufficient antibody would result in a weak SPR signal and narrow detection range, and too much antibody would affect the sensitivity of the immunosensor. Optimization was carried out to analyze the dilution of antibody I (1:5000, 1:2000, 1:1000, 1:500, 1:200, 1:100) without free RCT. As is shown in Figure 1c, obvious binding signals of RCT-BSA and antibody I were obtained, namely 78.2, 122.2, 234.4 RU with antibody I diluted at 1:1000, 1:500, and 1:200, respectively. Further assays would determine the suitable dilution.

As antibody I was competed—by RCT and RCT-BSA, pre-incubation of RCT and antibody I was performed for the prior binding between RCT and antibody I, and the pre-incubation time was optimized first. As is shown in Figure 1d–f, 30 min led to the best performance with all three dilutions of antibody I (1:1000, 1:500, and 1:200). Thus, we selected 30 min as the best pre-incubation time.

### 3.2. RCT Detection Using the Primary SPR Immunosesnor

Then the primary SPR immunosensor was developed for RCT detection in both PBS buffer and swine urine samples. RCT standards were spiked in PBS and pretreated swine urine samples with the final concentrations of 0.1, 0.3, 1, 2, 4, 8, 16, 32, and 64 ng/mL. RCT and antibody I were pre-incubated for 30 min, and then the pre-incubated mixtures were injected into the SPR channels and the consequent signals were collected. After each sample detection, the functionalized chip was regenerated with 50 mM NaOH flowing at 20 µL/min for 1 min.

**Figure 2:**
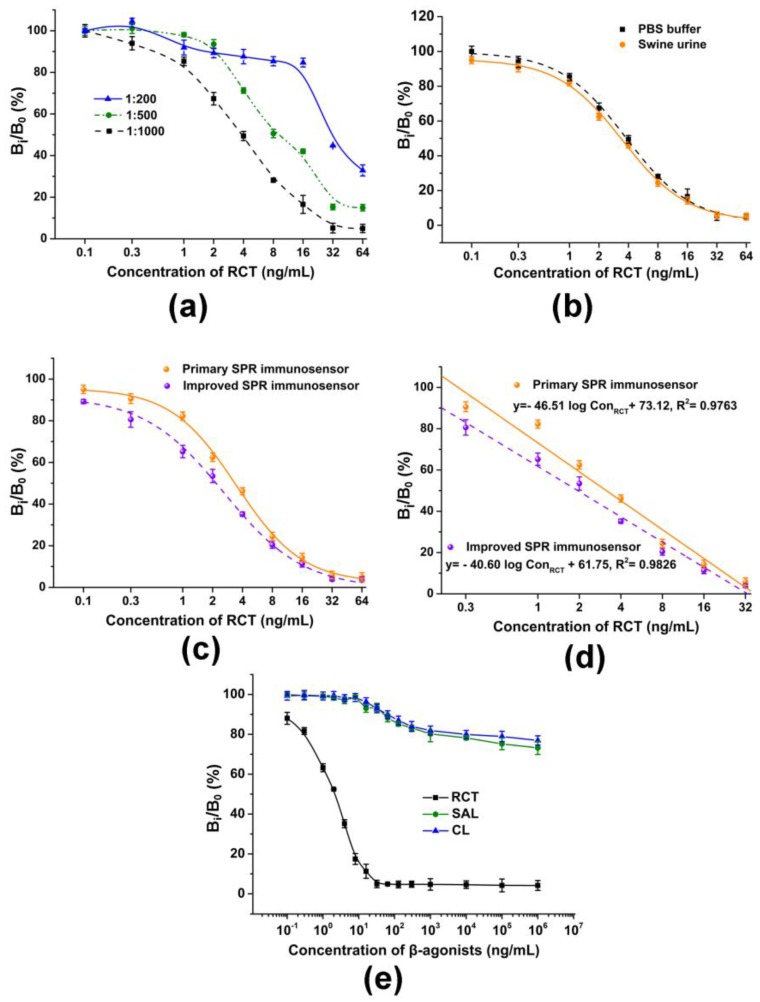
RCT detection in both PBS buffer and swine urine samples using the primary and improved SPR immunosensors. (**a**) Competition curves with different concentrations of antibody I using the primary SPR immunosensor; (**b**) Competition curves in PBS buffer and swine urine samples using 1:1000 antibody I with the primary SPR immunosensor; (**c**) Comparison of the primary and improved SPR immunosensors; (**d**) Calibration curves obtained from the primary and improved SPR immunosensors; (**e**) Specificity assay with different β-agonists. RCT, ractopamine; SAL, salbutamol; CL, clenbuterol. Data points are the average ± standard deviation (*n* = 3).

As shown in Figure 2a, a high concentration of antibody I (1:200, 1:500) resulted in a low sensitivity and narrow linear range, and the dilution of 1:1000 gave rise to a wider detection range and higher sensitivity. Thus, antibody I was diluted at the ratio of 1:1000 and used for RCT detection in real swine urine samples. Figure 2b shows that the primary SPR immunosensor performed well with the pretreated swine urine, with no significant matrix interference. However, the LOD was calculated to be 0.22 ng/mL, which did not prevail compared to the published methods in Table 1.

### 3.3. Improvement of the SPR Immunosensor with Antibody II

Antibody II which can bind specifically with antibody I was applied to improve the analytical performance of the primary SPR immunosensor. In detail, antibody II diluted at 1:9000 was injected simultaneously with the RCT/antibody I mixture and SPR signals were collected. As shown in Figure 2c, the inhibition curve of the improved SPR immunosensor shows a significant change compared with that of the primary SPR immunosensor. The relative binding percentage *(*B_i_/B_0_, %) decreased obviously to less than 90% when the RCT concentration was 0.1 ng/mL. Figure 2d demonstrates the obvious difference between the slopes of the two calibration curves obtained from the primary and improved SPR immunosensors, and the improved SPR immunosensor can improve the LOD to 0.09 ng/mL. The sensitivity was higher than most published methods for RCT detection (Table 1).

### 3.4. Specificity Assay and Recovery Study

Several common member β-agonist drugs were used to further validate the specificity of the SPR immunosensor. As is shown in Figure 2e, the immunosensor yielded highly specific recognition to RCT compared to other β-agonist drugs, with the concentration varying from 0.1 ng/mL to 1 mg/mL. Moreover, the IC50 (concentration corresponding to B_i_/B_0_ = 50%) of the RCT was calculated to be 1.96 ng/mL. In addition, the IC50 of SAL (salbutamol) and CL (clenbuterol) were both higher than 1 mg/mL. Cross-reactivity (%) was defined as IC50 (RCT)/IC50 (interferents) × 100. In addition, the cross-reactivity of the SPR immunosensor was calculated to be lower than 10^−4^, which shows the excellent specificity of the developed immunosensor.

The accuracy of the improved SPR immunosensor in detecting RCT in swine urine was then evaluated by the recovery rate and data reproducibility (relative standard deviation, RSD). Different RCT concentrations (0.3, 2.0, 16 ng/mL) were spiked in the negative swine urine sample, and the recovery rates were analyzed to be 101.8%–106.6%, and the RSD was lower than 8% (Table 2, *n* = 3).

### 3.5. Indirect Competitive ELISA and Comparison between the Two Immunosensors

**Figure 3:**
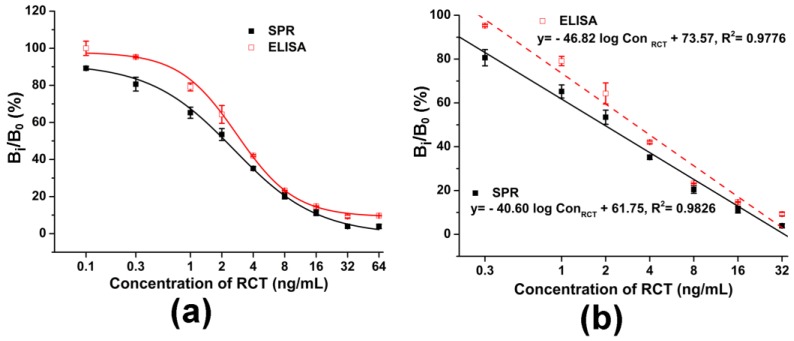
Comparison of the SPR immunosensor and the ELISA immunosensor for RCT detection in swine urine. (**a**) Dose-response curves obtained from the two immunosensors. (**b**) Calibration curves obtained from these two immunosensors. Data points are the average ± standard deviation (*n* = 3).

An indirect competitive ELISA was also developed for RCT detection in swine urine, with antibody I diluted at 1:1000 and HRP-labeled antibody II diluted at 1:9000. As shown in Figure 3, the ELISA immunosensor provided a similar linear range as the SPR immunosensor but did not get a significant relative binding signal until the RCT concentration increased to 1 ng/mL, and the LOD of the ELISA immunosensor was calculated to be 0.21 ng/mL (Figure 3a,b). Furthermore, Table 3 was used to further compare the two immunosensors. Although the two immunosensors provided similar linear detection ranges, the SPR immunosensor provided a lower LOD, and also required a smaller volume of reagents and less analysis time. Moreover, the whole analysis can be completed with automatic injection and signal generation.

## 4. Discussion

The SPR signal is induced by mass change and can achieve label-free detection; thus, SPR can be used as an alternative method to ELISA. The simplest way is to apply anti-RCT antibody to achieve label-free detection, just as was done in our primary SPR immunosensor. Herein, we further improved the SPR immunosenor with a secondary antibody, which led to a second competition process and improved the sensitivity of the immunosensor. SPR was mass-dependent and antibody II contributed to the mass of the molecules which bound to RCT-BSA. After the competition of RCT and RCT-BSA for antibody I, there would be two states of antibody I, one bound to RCT in solution in a relatively free state and the other bound to RCT-BSA in an immobilized state. Then antibody II was competed for by these “two kinds” of antibody I, leading to larger SPR signals, and thus improving the sensitivity of the immunosensor. Meanwhile, the immunosensor is more advantageous than other amplification methods as no chemical labels or materials were used in this assay.

The present design initially applying the antibody I/antibody II pair for small molecule detection can be further referred to for other small molecule detections in the future. In addition, as more and more recognition elements such as aptamers are being generated, aptamer/antibody pairs or aptamer/aptamer pairs can also be used in such a SPR immunosensor.

## 5. Conclusions

In conclusion, an indirect competitive label-free SPR immunosensor was developed and its performance was further improved by introducing a secondary antibody. The SPR immunosensor can provide a linear detection range of 0.3–32 ng/mL for RCT in swine urine samples, with a LOD of 0.09 ng/mL. Meanwhile, the SPR immunosensor showed high sensitivity, selectivity, and good stability. The SPR immunosensor was thoroughly compared with an indirect competitive ELISA, and the SPR immunosensor demonstrated significant advantages in terms of LOD, reagent consumption, analysis time, and operation automation. This kind of SPR immunosensor with a simple operation and a rapid response can be a promising sensing platform for real-time monitoring and quantitation of RCT or other small molecules in real-world samples.

## Figures and Tables

**Table 1:** Comparison of various analytical methods for RCT detection.

Analytical Methods	LOD (ng/mL)	Linear Range (ng/mL)	Sample	References
GC-MS	4	10–500	feed sample	[4]
LC-MS	1	Not available	Bovine muscle	[5]
Fluorescence immunoassay	0.1	1–10	swine tissue	[7]
Fluorescence immunoassay	1	2.3–50	pork	[8]
Nanoparticle immunoassay	0.34	1–40	pork	[9]
Aptamer-AuNPs-MIP	10	10–400	feed, beef	[14]
Electrochemical aptasensors	0.04	0.1–10	pork	[15]
SPR immunoassay	0.12	0.28–4.29	liver	[13]
SPR immunoassay	0.22	0.3–32	swine urine	This paper
Improved SPR immunoassay	0.09	0.3–32	swine urine	This paper

GC-MS, gas chromatography-mass spectrometry; AuNPs, gold nanoparticles; MIP, molecular imprinting polymers.

**Table 2:** Recovery study of the improved SPR immunosensor.

Spiked Concentration (ng/mL)	Measured Concentration (ng/mL, Mean ± SD)	Recovery (%, Mean)	RSD (%)
0.3	0.5161 ± 0.02532	103.2	4.906
2	2.1323 ± 0.1462	106.6	6.856
16	16.2898 ± 1.2731	101.8	7.815

**Table 3:** Thorough comparison of SPR immunosensor and the ELISA immunosensor.

		SPR	ELISA
**LOD**		0.09 ng/mL	0.21
**Linear Range**		0.3–32 ng/mL	0.3–32 ng/mL
**Solid matrix**	Matrix	CM5 chip	Microwells
	Reusability	Yes (>50 times)	No
**Reagents consumption**	RCT-BSA	110 μL in total	100 μL/sample
	Antibody I	80 μL/sample	50 μL/sample
	Antibody II/HRP-antibody II	80 μL/sample	100 μL/sample
	TMB	-	100 μL/sample
**Analysis time**	Immobilization time	11 min	Overnight
	Blocking time	7 min	2 h
	Competition time	4 min	45 min
	Signal amplification time	4 min	45 min
**Automation**		Yes	No
**Labeling**	Antibody I	No	No
	Antibody II	No	HRP
